# Bayesian spatiotemporal analysis for association of environmental factors with hand, foot, and mouth disease in Guangdong, China

**DOI:** 10.1038/s41598-018-33109-3

**Published:** 2018-10-11

**Authors:** Zhicheng Du, Wayne R. Lawrence, Wangjian Zhang, Dingmei Zhang, Shicheng Yu, Yuantao Hao

**Affiliations:** 1 Department of Medical Statistics and Epidemiology & Health Information Research Center & Guangdong Key Laboratory of Medicine, School of Public Health, Sun Yat-sen University, Guangzhou, 510080 China; 2 Key Laboratory of Tropical Diseases and Control of the Ministry of Education, Guangzhou, 510080 China; 3 Department of Environmental Health Sciences, School of Public Health, University at Albany, State University of New York, Rensselaer, 12144 USA; 4 Chinese Center for Disease Control and Prevention, Beijing, 102206 China

## Abstract

Hand, foot, and mouth disease (HFMD) remains a significant public health and economic burden in parts of China, particularly Guangdong Province. Although the association between meteorological factors and HFMD has been well documented, significant gaps remain in our understanding of the potential impact of environmental factors. Using county-level monthly HFMD data from China CDC and environmental data from multiple sources, we used spatiotemporal Bayesian models to evaluate the association between HFMD and environmental factors including vegetation index, proportion of artificial surface, road capacity, temperature and humidity, and assessed the spatial and temporal dynamic of the association. Statistically significant correlation coefficients from −0.056 to 0.36 (all *P* < 0.05) were found between HFMD incidence and all environmental factors. The contributions of these factors for HFMD incidence were estimated to be 16.32%, 12.31%, 14.61%, 13.53%, and 2.63%. All environmental factors including vegetation index (Relative Risk: 0.889; Credible Interval: 0.883–0.895), artificial surface (1.028; 1.022–1.034), road capacity (1.033; 1.028–1.038), temperature (1.039; 1.028–1.05), and relative humidity (1.015; 1.01–1.021) were statistically retained in the final spatiotemporal model. More comprehensive environmental factors were identified as associating with HFMD incidence. Taking these environmental factors into consideration for prevention and control strategy might be of great practical significance.

## Introduction

Hand, foot, and mouth disease (HFMD) remains a major public health concern in China, where over two million children are affected annually^1^. Guangdong is one of the most affected provinces in China where the incidence of HFMD exceeds 30 cases per 10,000 annually, and in recent years accounted for approximately 15% of total cases in China^2^. Even though vaccines have entered the market substantially reducing Enterovirus 71 (EV71), which is one of the major causative pathogens of HFMD, in certain areas of China the epidemic remains. Pathogens other than EV71 have been confirmed to be causative for HFMD, such as Coxsackievirus A16 (CVA16) and CVA6^3^. HFMD caused by these pathogens have become increasingly dominant. While pathogen characteristics such as the activity and type of pathogens at the point of infection are more likely directly related to HFMD onsets, environmental factors are potentially associated with HFMD infections^4^.

Among various environmental factors, weather variables are investigated more for their effect on HFMD hazards. A nationwide study in China suggested that the seasonal patterns of HFMD were associated with precipitations, sunshine, temperature, and air pressure^5^. However, previous climatic variables do not explain the complexity of HFMD seasonality across China^5^. Zhao *et al*.^6^ and Gou *et al*.^7^ reported that high temperature could increase the risk of HFMD in Huainan and Gansu, China. In our previous study, we observed a higher risk of HFMD when temperatures were greater than 24.85 °C and relative humidity within 80.59–82.55%^8^.

Nonetheless, the relationship between disease and environmental factors are complex and other environmental factors besides climate factors may contribute to the HFMD epidemics. Modifiable environmental factors were reported to be a reason for approximately 23% of global disease burden, where over one-third are among children^9^. Climatic factors primarily contribute to temporal variation for a relatively small geographic area, while other environmental factors would explain more the spatial variation. However, a question arises whether environmental factors besides climate factors have been investigated on human health, especially infectious diseases?

Multiple factors including ecological, environmental, agricultural, industrial, and many others could precipitate disease emergence^10^. A spatial analysis by Wayant *et al*. showed that malaria rates were associated with the changing land cover, particularly due to deforestation^11^. However, reforestation which could be reflected by vegetation index and land cover was found to be a probable reason for the emergence of Lyme disease in the United States and Europe^12^. Occasionally, an infection is brought to a new place through travel and commerce^13^. Similarly, rural urbanization would allow infections arising in isolated rural areas to reach larger populations. For this reason, a question emerges on whether these factors are attributed to HFMD transmission.

Currently, limited studies have examined other environmental factors. A recent publication concluded that the normalized difference in vegetation index (NDVI) has an important role as a predictor to HFMD incidence in Shandong, China^14^. However, they reported the contribution to psudo R^2^ of NDVI rather than an unambiguous statistics such as the relative risk (RR) or the solpe (β). Therefore, we can not conclude a total positive or negative effect of NDVI on HFMD. A study by Cao and colleagues^15^ checked the relationship between HFMD epidemics in Shenzhen and road density, aerosol optical depth, and NDVI. However, none of the above factors remained in the final linear regression. The simple linear model most likely did not have enough statistical power to observe an association.

To provide a more comprehensive understanding of HFMD associated environmental factors, we applied a Bayesian spatiotemporal model to identify and develop a prediction model based on available vital factors including temperature (T), relative humidity (RH), NDVI, land cover (proportion of artificial surface, AS), and road capacity (RC).

## Results

### Descriptive analysis

**Figure 1:**
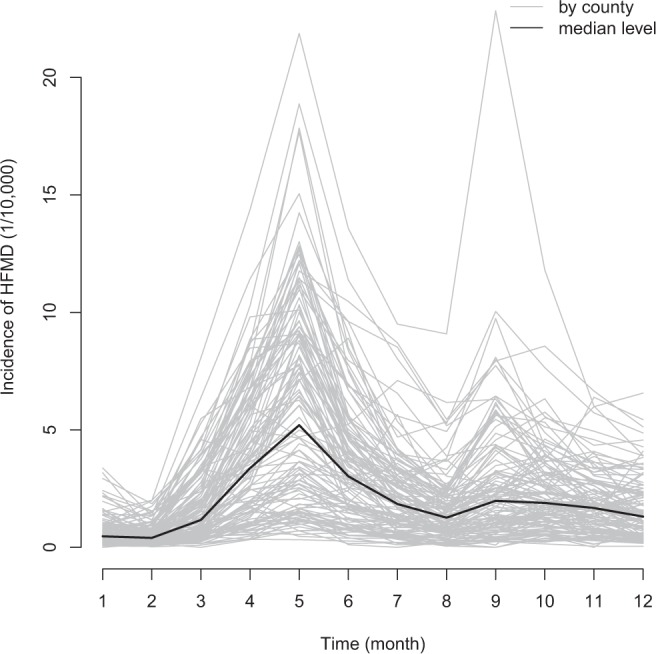
Monthly trend line of HFMD incidence for each county and the median level in Guangdong.

The median monthly HFMD incidence and environmental factors (T, RH, NDVI, RC) by county were 1.51 (per 10,000 persons), 233.68 (0.1 °C), 79.19 (%), 6053.13, 3.77 (%), and 57.01, respectively. The highest incidence during the study period was 22.84 per 10,000 persons. Generally, monthly incidence for counties showed similar trend with a major peak in May and a smaller peak in September (Table 1 and Fig. 1).

**Table 1:** Descriptive statistics of the monthly HFMD incidence and environmental factors by county in Guangdong.

Variable	Range	Mean (SD)	Median (Quartile)
Incidence (per 10,000 persons)	(0,22.84)	2.4(2.67)	1.51(0.64,3.17)
T (0.1 × °C)	(38.94,304.11)	216.6(61.73)	233.68(162.26,272.85)
RH (%)	(59.55,100)	78.94(5.5)	79.19(75.57,82.27)
NDVI	(1447.18,7992.3)	5745.78(1496.75)	6053.13(4722.39,6969.02)
Cultivated land (%)	(0,74.5)	27.09(15.72)	25(15.83,36.36)
Forest (%)	(0.18,81.59)	44.75(22.44)	46.86(24.53,64.85)
Grassland (%)	(0,44.09)	7.27(6.13)	6.37(3.28,9.65)
Shrubland (%)	(0,15.99)	1.4(2.94)	0(0,0.98)
Wetland (%)	(0,5.1)	0.11(0.56)	0(0,0.01)
Water bodies (%)	(0.09,42.95)	7.01(8.63)	3.8(1.11,9.51)
Artificial surfaces(%)	(0.27,79.29)	11.97(16.48)	3.77(1.42,15.11)
Bareland (%)	(0,0.72)	0.01(0.07)	0(0,0)
RC	(8.98,476.96)	63.52 (54.42)	57.01(26.82,81.34)

*Abbreviation: HFMD, hand, foot, and mouth disease; T, temperature; RH: relative humidity; NDVI, normalized difference vegetation index; AS, artificial surface; RC, road capacity; SD: standard deviation.

### Crude correlation

**Figure 2:**
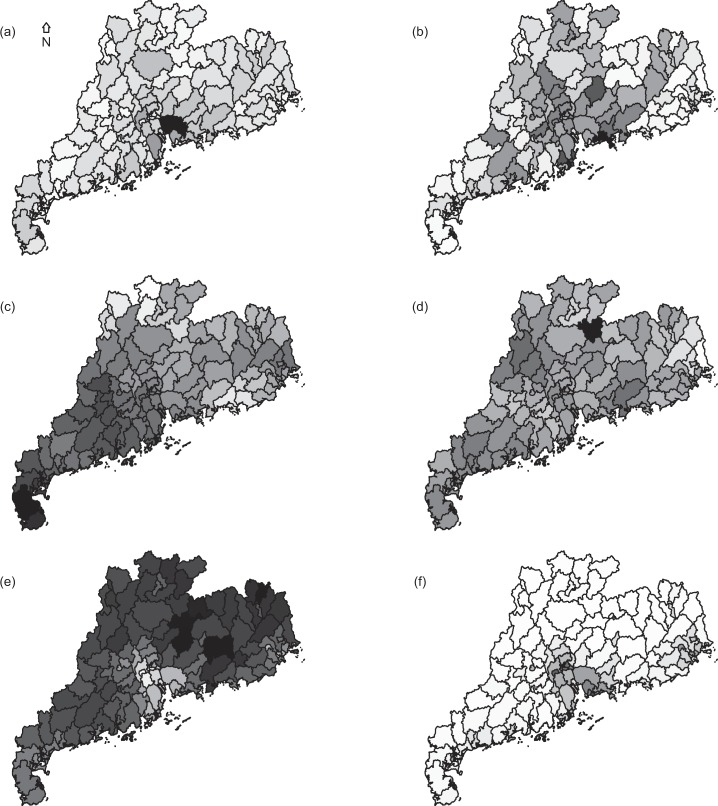
Maps for the median value of monthly HFMD incidence and associated environment factors for each county. (**a**) incidence of hand, foot, and mouth disease; (**b**) average temperature; (**c**) average relative humidity; (**d**) normalized difference vegetation index; (**e**) artificial surface; (**f**) road capacity. This map was downloaded from OpenStreetMap (The cartography in the OpenStreetMap map tiles is licensed under CC BY-SA (www.openstreetmap.org/copyright, © OpenStreetMap contributor). The licence terms can be found on the following link: http://creativecommons.org/licenses/by-sa/2.0/) and processed by and R version 3.4.3 (R Core Team, Vienna, Austria, 2017, https://www.R-project.org).

The absolute value of correlation coefficients from the spearman test between HFMD incidence and environmental factors ranged from 0.056 to 0.36 (all P < 0.05). Of the 8 types of land cover, artificial surfaces received the maximum correlation coefficient (Results for other types of land cover are not shown). The maps for the median value of HFMD incidence and environmental factors in May also suggested an association between them (Table 2 and Fig. 2). The varied seasonal variations were observed from the temporal distributions for the median value of HFMD incidence and environmental factors (Supplementary Figure S1).

**Table 2:** Spearman correlation coefficients between HFMD incidence and environmental factors.

	Incidence	T	RH	NDVI	AS
T	0.36	—	—	—	—
RH	0.087	−0.064^¶^	—	—	—
NDVI	−0.056	0.344	−0.176	—	—
AS	0.266	0.098	0.021^¶^	−0.69	—
RC	0.161	0.006^¶^	−0.068^¶^	0.084	0.143

*Abbreviation: T, temperature; RH, relative humidity; NDVI, normalized difference vegetation index; AS, artificial surface; RC, road capacity.

^¶^Not statistically significant, P > 0.05.

### Spatial autocorrelation

The estimated Moran’s I statistics among eleven of the twelve months were statistically associated (P < 0.05), ranging from 0.1 to 0.25 (Table 3).

**Table 3:** Moran’s I statistic of HFMD cases for each month in Guangdong.

Month	Statistic	P value	Month	Statistic	P value
Jan	0.07	0.09	Jul	0.14	0.01
Feb	0.1	0.03	Aug	0.15	<0.001
Mar	0.12	0.02	Sep	0.17	<0.001
Apr	0.19	<0.001	Oct	0.18	<0.001
May	0.25	<0.001	Nov	0.11	0.02
Jun	0.23	<0.001	Dec	0.15	0.01

### Model development

**Figure 3:**
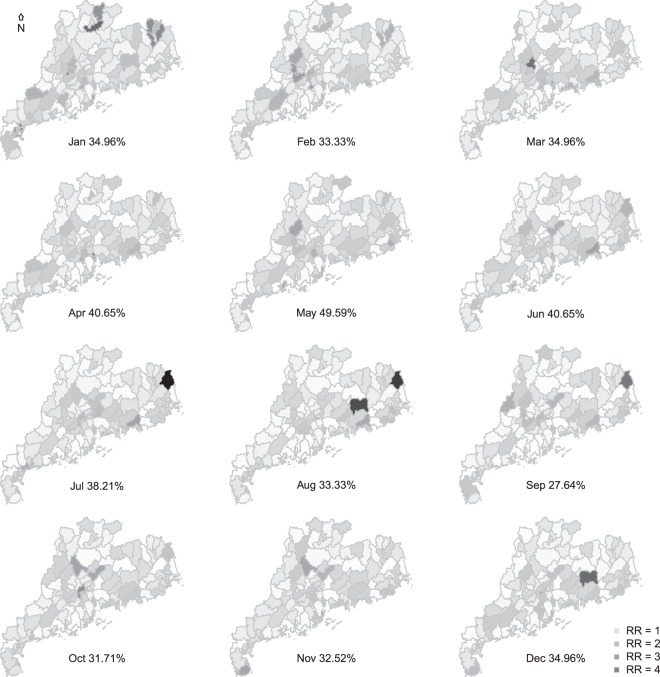
The spatiotemporal distribution of HFMD risk. The proportion of counties with RR >1 for each month noted under the map. This map was downloaded from OpenStreetMap (The cartography in the OpenStreetMap map tiles is licensed under CC BY-SA (www.openstreetmap.org/copyright, © OpenStreetMap contributor). The licence terms can be found on the following link: http://creativecommons.org/licenses/by-sa/2.0/) and processed by and R version 3.4.3 (R Core Team, Vienna, Austria, 2017, https://www.R-project.org).

The contribution (Reduction in DIC) of environmental factors (e.g., RH, T, AS, RC, and NDVI) were 2.63%, 13.53%, 12.31%, 14.61%, and 16.32%. All above facotrs were retained in the final model. The RR were 1.015, 1.039, 1.028, 1.033, and 0.889, respectively, with all credible intervals rejecting the null hypothesis (Tables 4 and 5). All spatiotemporal parameters including the variance of spatial autocorrelation (*τ*^2^), spatial and temporal autoregressive parameter (*ρ*_*S*_ and *ρ*_*T*_), and variance of localized spatial autocorrelation (*τ*_*w*_^2^) were statistically detected with a much larger *τ*_*w*_^2^ than the *τ*^2^ (Table 6). The spatiotemporal distribution showed an irregular trend with county-level pattern varying monthly (Fig. 3). The proportion of counties with RR >1 reached the highest level (49.59%) in May. Some counties with high RR (darker color) occasionally appeared in the center or east of the study area.

**Table 4:** Applying Bayesian spatiotemporal model to fit HFMD incidence with environmental factors.

Model	DIC	Contribution (%)
CARadaptive	67780.72	reference
CARadaptive + RH	65995.02	2.63
CARadaptive + T	58610.25	13.53
CARadaptive + AS	59439.96	12.31
CARadaptive + RC	57874.96	14.61
CARadaptive + NDVI	56715.81	16.32

*Abbreviation: CARar, simple spatiotemporal model; CARadaptive, adaptive spatiotemporal model; T, temperature; RH, relative humidity; NDVI, normalized difference vegetation index; AS, artificial surface; RC, road capacity; DIC, deviance information criterion, the smaller the better.

**Table 5:** The estimated relative risks of the environmental factors in the final Bayesian spatiotemporal model.

Variable	RR	2.5%	97.5%
RH	1.015	1.010	1.021
T	1.039	1.028	1.050
AS	1.028	1.022	1.034
RC	1.033	1.028	1.038
NDVI	0.889	0.883	0.895

*Abbreviation: RH, relative humidity; T, temperature; AS, artificial surface; RC, road capacity; NDVI, normalized difference vegetation index; RR, relative risk.

**Table 6:** The estimated spatiotemperal parameters in the final Bayesian spatiotemporal model.

Parameter	Median	2.5%	97.5%
*τ* ^2^	0.392	0.35	0.442
*ρ*_*S*_	0.283	0.192	0.384
*ρ*_*T*_	0.82	0.785	0.855
*τ*_*w*_^2^	156.235	117.567	202.09

*τ^2^, the variance of spatial autocorrelation; *ρ*_*S*_, *ρ*_*T*_, spatial and temporal autoregressive parameter; *τ*_*w*_^2^, the variance of localized spatial autocorrelation.

### Validation

**Figure 4:**
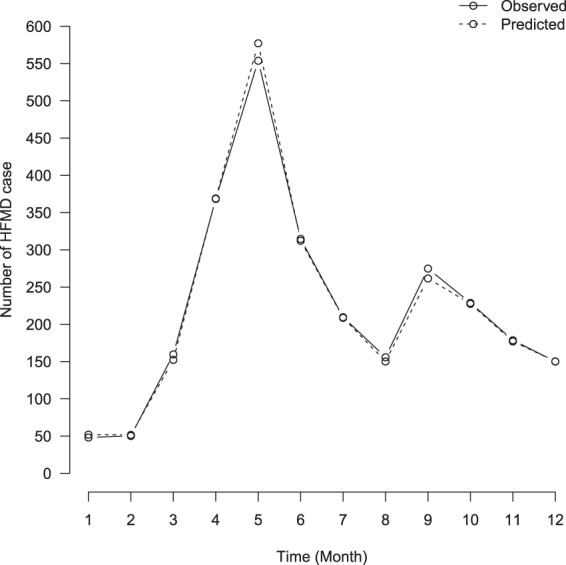
The observed and predicted monthly average number of HFMD cases by using the final spatiotemporal Bayesian model.

The final model had a smaller sMAPE value of 13.853% (19.774% for the testing dataset in 2011), which was significantly smaller than 100%. The county-level sMAPEs range varied from 1.093% to 90.506% (2.358% to 75.659% for the testing dataset, Supplementary Table S1). The monthly trend line for observed and predicted number of HFMD cases almost matched (Fig. 4 and Supplementary Figure S2). The convergence diagnostics for MCMC algorithms were presented as trace plots (Supplementary Figure S3). The density of trace for all parameters presented normal distribution.

## Discussion

Our study of more than 0.33 million cases of HFMD reported to the national surveillance system during 2012 in Guangdong, China provides a comprehensive association for environmental factors on HFMD epidemics. The highest monthly incidence during the study period was 22.84 per 10,000 persons. We observed the crude correlation coefficients, spatial distribution (maps), and temporal distribution (time-series plots) between environmental factors and HFMD incidence. All environmental factors indicated a potential association for disease transmission with statistically significant coefficients. The seasonal autocorrelation and spatial autocorrelation were shown and validated in the time series plots and the Moran’s I statistic. A Bayesian spatiotemporal model was used to fit these two autocorrelations. We estimated the contribution (i.e., the explained variation of DIC) of each environmental factor. All environmental factors in the final Bayesian spatiotemporal models gained statistical association, indicating a relatively comprehensive association for HFMD with environmental factors was established.

The association between environmental factors used in the present study with infectious diseases were widely studied. For HFMD, the relative humidity and temperature were considered in prior studies and in the present^8,16^. Most of these studies provided quantitative evidence that the number of HFMD cases increased significantly with rising average temperature and relative humidity. Satellite data (e.g., land-use, NDVI, and El Niño-Southern Oscillation (ENSO)) has also been used to explain the transmission of other infectious diseases, such as malaria in Thailand, Afghanistan, and Korea; dengue in Indonesia; avian influenza in Indonesia; and seasonal influenza in New York, Arizona, and Hong Kong^17^. This suggest that land-use and NDVI affect the risk of disease via vector breeding habitat. By contrast, negative association (RR < 1) between NDVI and HFMD risk was observed in our study. Li *et al*.^14^ and Cao *et al*.^15^ concluded a negative correlation as well between NDVI and HFMD risk.

Environmental factors that potentially affect disease include virus survivorship, transmission efficiency, and host susceptibility. In the present study, higher levels of relative humidity and temperature were positively associated with HFMD epidemics, potentially enhancing virus activity of the disease. The higher level of road capacity might improve transmission efficiency by increasing the interaction among susceptible individuals. Rather than the spatiotemporal model, similar crude relationship was observed using the Spearman’s correlation coefficient^15^. We also observed that HFMD epidemics were positively linked to the proportion of artificial surface. Curran *et al*. also suggested links between land cover and the disease carried by vectors^18^. Higher levels of artificial surface indicates that elevated degree of urbanization resulted in weakness to health services among immigrant workers. A potential mediated factor for environmental factors is environmental pollution. Environmental pollution, particularly air pollution was impacted by road capacity (positive), artificial surface (positive), and vegetation index (negative)^19^. Moreover, these pollutions improve transmission efficiency of the virus and increases host susceptibility to disease.

The spatial and temporal autocorrelation of HFMD epidemics shown in our results were also validated previously. Ma *et al*. revealed the spatiotemporal pattern of HFMD by reviewing 73 relevant studies^4^. In our prior study on the national level in China also observed the spatial and temporal autocorrelation patterns of HFMD^8^. Overall, the methods and results for detecting spatial and temporal autocorrelation used in this study were consistent with previous studies.

The present study stood out from prior studies by its strengths. First, we included more aspects of environmental factors than previous studies that considered only climate factors. To the best of our knowledge, this is the first study to establish this type of comprehensive model for HFMD. Second, because environmental factors were collected from government sectors (e.g., NASA) and recognized programs (e.g., OpenStreetMap), we can be assured of data reliability. Moreover, the Bayesian spatiotemporal model has been widely used with an acceptable goodness of fit with a low sMAPE in the fitting and testing datasets (13.853% and 19.774%), and a considerable convergence diagnostic for MCMC algorithms.

We must also note a few limitations. First, although we observed a comprehensive association between HFMD and environmental factors, the detailed mechanism is not well understood. Second, some environmental factors were collected at periods different from the disease, potentially resulting in statistical analyses uncertainty. However, environmental factors would change in a slow manner, where the relative level of environmental factors among counties might be consistent over years. For this reason, the current available information on environmental factors is most likely a reasonable representation. In addition, there might be a complicated association (e.g., non-linear or interactive) between HFMD risk and environmental factors. We applied the ‘Log’ link function to handle part of the non-linear relationship, and the Bayesian spatiotemporal model used in our study is currently an available model to cope with both spatial and temporal effects.

## Conclusion

This study provides quantitative evidence for environmental effects on HFMD in Guangdong using a more comprehensive established model. The contributions of each factors were 16.32% (NDVI), 14.61% (RC), 13.53% (T), 12.31% (AS), and 2.63% (RH), respectively. Of the association between environmental factors and HFMD, only NDVI was a protective factor. This information could be helpful in guiding health resource allocations and developing public health preparedness and intervention strategies.

## Methods

### Ethics statement

This study was based on official HFMD surveillance data in China. Analyses were conducted at aggregate level and no confidential information was involved. The research study protocol was approved by the Institutional Review Board at Sun Yat-sen University School of Public Health (No. 201415). All methods were performed in accordance with the principles of the Declaration of Helsinki.

### Study area

Guangdong Province, situated north latitude 20°21′ N to 25°51′ N and east longitude 109°65′ E to 117°31′ E, has a population of 104 million as reported in the 2010 census. According to the Annual Statistical Report of Guangdong Province (http://www.gdstats.gov.cn/tjsj/gdtjnj/), 123 counties can largely be divided into four parts, (i.e., Pearl River Delta Region [around the center of the province], Eastern Region, Western Region and Mountainous Region). These four regions are in different stages of social and economic transition. The Pearl River Delta Region is the most industrialized area of Guangdong, where region has a higher HFMD burden compared to the other three areas. For example, the accumulative incidence from 2009 to 2012 in Pearl River Delta Region was 39 per 10,000, much greater than the other regions in Guangdong during the same period (Eastern Region: 7/10,000; Western Region: 12/10,000; Mountainous Region: 14/10,000).

### Data collection

Monthly number of HFMD cases for each county in 2012 were obtained from the Data-center of China Public Health Science (http://www.phsciencedata.cn/Share/en/index.jsp), China Center for Disease Control and Prevention (China CDC). A total of 0.33 million HFMD cases out of a population of 105.94 million were confirmed by the guidelines for diagnosis and treatment of HFMD, and were reported to the national surveillance system^1,20^.

Monthly meteorological data including average temperature (T) and average relative humidity (RH) for each county in 2012 were downloaded from China Meteorological Data Sharing Service System (http://cdc.cma.gov.cn).

Monthly normalized difference vegetation index (NDVI) for Guangdong province in 2012 were downloaded from the moderate resolution imaging spectroradiometer (MODIS, https://modis.gsfc.nasa.gov/data/). ArcMap version 10.2 (Environment System Research Institute, Redlands, California, USA) was used to extract the NDVI for each county.

Land cover data for Guangdong province in 2010 were downloaded from China National Catalogue Service for Geographic Information (http://www.webmap.cn/). Land cover data included cultivated land (lands used for agriculture, horticulture, and gardens); forest (lands covered with trees, with vegetation cover over 30%); grassland (lands covered with natural grass with cover over 10%); shrub land (lands covered with shrubs with cover over 30%); wetlands (lands covered with wetland plants and bodies of water); bodies of water (bodies of water in land area); artificial surfaces (AS, lands modified by human activities); and bareland (lands with vegetation cover lower than 10%). 2010 national land cover data was used, as 2012 was unavailable. We calculated the proportion of different types of land cover for each county using ArcMap. Only the AS was included in the model fitting to avoid the multicollinearity among the 8 types of land cover.

Roads data for Guangdong province on March 2, 2017 were downloaded from OpenStreetMap (OSM, www.openstreetmap.org). Since this data is generally updated every day, 2017 data were used to represent 2012 in our study. Different types of roads including motorway, primary roads, secondary roads, and tertiary roads and their length were extracted for each county using ArcMap. According to the Chinese Technical Standard of Highway Engineering (JTG B01-2014)^21^, different types of roads have different traffic capacity (motorway: 15,000 vehicles per day; primary roads: 15,000; secondary roads: 10,000; and tertiary roads: 4,000). For this reason, we calculated the road capacity (RC) for different road types by multiplying the traffic capacity and their length. Although OSM is often more up-to-date and of a higher quality than other commercial maps, we used JOSM (https://josm.openstreetmap.de/, a quality assurance tool for better OSM data) to check and fix invalid data automatically.

County level shapefile (.shp) of Guangdong province was obtained from OpenStreetMap.

### Data analysis

A province-wide ecological study was conducted. First, we calculated the basic descriptive statistics (e.g., range, mean, standard deviation (SD), median, and quartile) for the incidence of HFMD and associated environmental factors including T, RH, NDVI, land cover, and RC.

Crude relationship between HFMD incidence and environmental factors were checked using the spearman correlation coefficient and disease mapping. Since the number of land cover variables were relatively more than others, we selected the type with maximum correlation coefficient. The median values of monthly HFMD incidence and environmental factors for each county were mapped out.

Time series plot and Moran’s I statistic^22^ were used to measure the temporal and spatial autocorrelation for the distribution of HFMD epidemic, respectively.

Before fitting spatiotemporal Bayesian models, all environmental factors, except T, were standardized to eliminate the influence caused by different dimensions. In order to handle the non-linear effect, T was divided into two categories (<25 °C and ≥25 °C) with the cut-off point of 25 °C^23^. Initially, we estimated the univariate effect for environmental factors. From there, a manual backward selection process was used to determine the final multivariable model to fit the HFMD epidemic. In this procedure, environmental factors with 95% credible interval (CI) covering the null hypothesis would be excluded. Additionally, we calculated the deviance information criterion (DIC) and the proportion of variation explained (Contribution) for the estimation of model and factors. The differences in information criterion versus the null model are very important and useful to the model selection and multi-model inference^24^. Trace plots were used to assess the convergence of markov chain monte carlo (MCMC) simulation in the Bayesian model fitting (Supplementary Figure S4).

Finally, we validated the final model by comparing the predicted and observed value. The symmetric mean absolute percentage error (sMAPE) was used to assess the fitting performance.

As we used aggregated data, interaction analysis was not applicable. All data analysis was conducted in R version 3.4.3 (R Core Team, Vienna, Austria), using the ‘base’, ‘GISTools’, ‘gstat’, ‘maptools’, ‘RColorBrewer’, ‘psych’, ‘spdep’, and ‘CARBayesST’ packages.

### Bayesian spatiotemporal model

As reported by the previous studies^4^, a spatiotemporal model would be suitable to the HFMD epidemics with spatial autocorrelation and seasonality. We applied a Bayesian spatiotemporal generalized linear mixed model with a spatiotemporal autoregressive process that has an adaptive autocorrelation structure. This model was proposed by Rushworth *et al*.^25^, and is an extension of the Bayesian spatiotemporal generalized linear mixed model with a spatiotemporal autoregressive process to allow for local spatial heterogeneity (adaptive smoothing) rather than global smoothing. Bayesian spatiotemporal model can be represented in a hierarchical structure as specified in the equation below.

$${Y}_{kt}\sim {\rm{Poisson}}({\mu }_{kt}),\,\mathrm{ln}({\mu }_{kt})=\beta \ast {{X}_{kt}}^{{\rm{T}}}+{O}_{kt}+{\psi }_{kt}$$

Lt *Y* and *O* denote the observed and expected number of HFMD cases, *k* and *t* denote the units of county and month. For each *k* = 1, …, 123 and *t* = 1, …, 12. The regression parameters of the vector of covariate *X* are denoted by *β*. The relative risk (RR) of *X* can be calculated as *e*^*β*^. The *ψ*_*kt*_ term is a latent component for county unit *k* and time period *t* encompassing one or more sets of spatiotemporally auto correlated random effects. Practically, the temporal variation can be extracted from *ψ*_*kt*_ by computing the temporal marginal effect. The autoregressive random effects structure of the model is specified by “ *ψ*_*kt*_ = *Φ*_*kt*_, *Φ*_*t*_|*Φ*_*t*−1_ ~ N(*ρ*_*T*_ * *Φ*_*t*−*1*_, *τ*^2^ * Q(W, *ρ*_*S*_)^−1^) for *t* = 2,…,12, *Φ*_*1*_ ~ N(0, *τ*^2^ * Q(W, *ρ*_*S*_)^−1^), *τ*^2^ ~ Inverse-Gamma(*a*, *b*), *ρ*_*S*_, *ρ*_*T*_ ~ Uniform(0,1)”. *Φ*_*t*_ is the vector of random effects for time period *t*, which evolve over time through a multivariate first order autoregressive process with temporal autoregressive parameter *ρ*_*T*_. The temporal autocorrelation is thus induced by the mean *ρ*_*T*_ * *Φ*_*t-1*_, while spatial autocorrelation is induced by the variance *τ*^2^ * Q(W, *ρ*_*S*_)^−1^. The corresponding precision matrix Q(W, *ρ*_*S*_) was proposed by Leroux *et al*.^26^. W is the symmetric nonnegative 123 × 123 neighborhood matrix, controlling the spatial autocorrelation. Different from the simple conditional autoregressive (CAR) model, this model allows for localized spatial autocorrelation by allowing spatially neighboring random effects (*τ*_*w*_^2^ ~ Inverse-Gamma(*a*, *b*)) to be correlated (inducing smoothness) or conditionally independent (no smoothing). The default values for the inverse-gamma prior for *τ*^2^ and *τ*_*w*_^2^ are *a* = 1, *b* = 0.01. The package ‘CARBayesST’ allows for estimating the unknown parameters including *τ*^2^, *ρ*_*S*_, *ρ*_*T*_, and *τ*_*w*_^2^ using MCMC method with Gibbs sampling. A single Markov chain was run for 110,000 iterations with a 10,000 burn-in period and subsequently thinned by 10 to reduce the autocorrelation in this study.

## Electronic supplementary material

Supplementary Tables and Figures

## Data Availability

The environmental data analysed during the current study are available from the sources described in ‘Data collection’. The HFMD data included in the study are available from China CDC (http://www.phsciencedata.cn/Share/en/index.jsp) but restrictions apply to the availability of this data, which were used under license, and so are not publicly available.
