# Supplementary material for: Bayesian spatiotemporal analysis for association of environmental factors with hand, foot, and mouth disease in Guangdong, China
Source: Sci Rep. 2018 Oct 11;8:15147. doi: 10.1038/s41598-018-33109-3 (PMC6181968; doi:10.1038/s41598-018-33109-3)
Supplement: Supplementary file 1 — Supplementary Tables and Figures [file 41598_2018_33109_MOESM1_ESM.pdf]

Bayesian spatiotemporal analysis for association of environmental factors with hand, foot and mouth disease in Guangdong, China

Zhicheng Du, Wayne R. Lawrence, Wangjian Zhang, Dingmei Zhang, Shicheng Yu, Yuantao Hao

Supplementary Tables

Table S1. The county-level symmetric mean absolute percentage errors.

COUNTY	SMAPE_TRAIN	SMAPE_TEST	COUNTY	SMAPE_TRAIN	SMAPE_TEST
baiyun	15.43423122	18.43112458	luoding	56.17654365	40.2619278
baoan	16.6751775	18.91884259	luogang	7.608855635	20.3237483
boluo	31.84834673	29.19954814	luohu	6.044597001	15.58347227
chancheng	3.270536227	9.166751001	maogang	6.624426632	13.7357943
chaoan	6.357338154	8.376517275	maonan	6.766247781	10.08182864
chaonan	1.151419863	11.05249767	mazhang	5.483973174	25.902658
chaoyang	1.093827339	4.902993753	meijiang	2.156167737	34.62951115
chenghai	14.02052465	13.7822119	meixian	4.497490949	11.91059363
chikan	14.30593652	10.04693787	nan'ao	90.50592211	38.88184588
conghua	29.01669997	8.303224612	nanhai	25.24823936	20.76897676
dapu	3.059870271	21.01406243	nansha	8.938142833	13.46033037
deqing	12.9461735	32.55773794	nanshan	21.72400437	37.07191216
dianbai	3.056559601	2.358281079	nanxiong	17.02058313	21.57915385
dinghu	5.017856701	18.58110861	panyu	14.19147246	8.102471559
dongguan	20.56667776	24.32535943	pengjiang	7.848819291	14.1010826
dongyuan	9.590246815	26.00464361	pingyuan	15.53522409	75.65906224
doumen	15.66929175	21.56818185	potou	3.379411212	13.28031724
duanzhou	11.99814503	18.08099926	puning	69.00552265	55.09665327

enping	10.60250007	17.31439092	qingcheng	7.627877783	27.33634492
fengkai	5.278663194	22.60577398	qingxin	8.794936427	11.7413163
fengshun	6.86561541	24.03433171	qujiang	3.827123973	19.78239136
fogang	7.081422717	8.624251532	raoping	5.687575378	14.85716242
futian	61.35711398	67.39048994	renhua	21.02715042	53.61258956
gaoming	8.338236439	12.33348938	rongcheng	4.373306767	11.89988898
gaoyao	1.899043365	13.63345604	ruyuan	30.915925	9.631424395
gaozhou	5.637513408	39.77068171	sanshui	2.769849479	16.88605427
guangning	9.05420902	42.05651532	shenwei	3.589360787	15.9504326
haifeng	5.450899805	4.049940417	shixing	24.32371161	73.61957373
haizhu	32.13283815	13.88596034	shunde	8.310419539	13.43046091
haojiang	4.125524657	7.301092157	sihui	7.862749916	13.64205282
heping	5.8926666	6.063228708	suixi	16.62274604	12.70325414
heshan	7.742441234	13.26322628	taishan	18.01139863	8.350878503
huadu	47.50876222	24.165978	tianhe	13.98357444	12.41821339
huaiji	13.04125864	16.21054327	wengyuan	3.675833949	7.937748142
huangpu	4.486779613	9.936828505	wuchuan	3.591912974	3.987174136
huazhou	16.29813337	16.80707427	wuhua	21.93348748	10.11580602
huicheng	18.43543232	22.17903992	wujiang	7.426664201	8.978492395
huidong	14.45000231	26.94781448	xiangqiao	10.25846402	11.5507932
huilai	3.170425221	6.946526564	xiangzhou	34.41943036	26.6157557
huiyang	13.36581774	22.60167013	xiashan	2.146119386	2.883780767
jiangcheng	10.63712094	23.39719323	xinfeng	20.55599124	21.84404133
jianghai	10.98386789	25.59338708	xingning	14.13038336	12.99565577
jiaoling	27.4776315	17.30311123	xinhui	29.54186749	19.64533574
jiedong	4.599868873	10.88187452	xinxing	17.4440536	14.94259031
jiexi	2.520975398	13.76367902	xinyi	7.426705036	15.94356455
jinping	3.544756256	6.133310701	xuwen	5.300582851	5.958009177
jinwan	5.671959515	13.60803003	yangchun	10.95807423	10.32898902

kaiping	12.60163812	11.37593367	yangdong	3.590307236	5.486692452
lechang	5.478988102	11.39695923	yangshan	8.220171713	28.64585422
leizhou	4.69483048	11.52861454	yangxi	4.334701546	55.0844333
lianjiang	23.77309057	30.11323276	yantian	7.91244086	15.30214051
liannan	15.97403667	21.20616959	yingde	9.516645002	17.14089239
lianping	17.29563959	17.67173518	yuancheng	9.665074048	15.19795021
lianshan	42.16404301	72.98587451	yuexiu	11.7155777	15.17552229
lianzhou	6.687854146	9.66097239	yun'an	19.17829005	30.09158647
liwan	7.573550335	11.60786752	yu'nan	5.050645824	8.336175438
longchuan	13.07749786	30.3282556	yuncheng	7.73685717	9.659842097
longgang	68.59242384	66.32641784	zengcheng	21.74932225	30.14430157
longhu	3.076225046	6.061120301	zhenjiang	6.078584342	17.31727035
longmen	9.664100916	3.558341178	zhongshan	15.52797547	22.07492589
lufeng	1.390485275	22.51960018	zijin	16.4415608	24.05734434
luhe	7.102783407	6.695766672			

Supplementary Figures

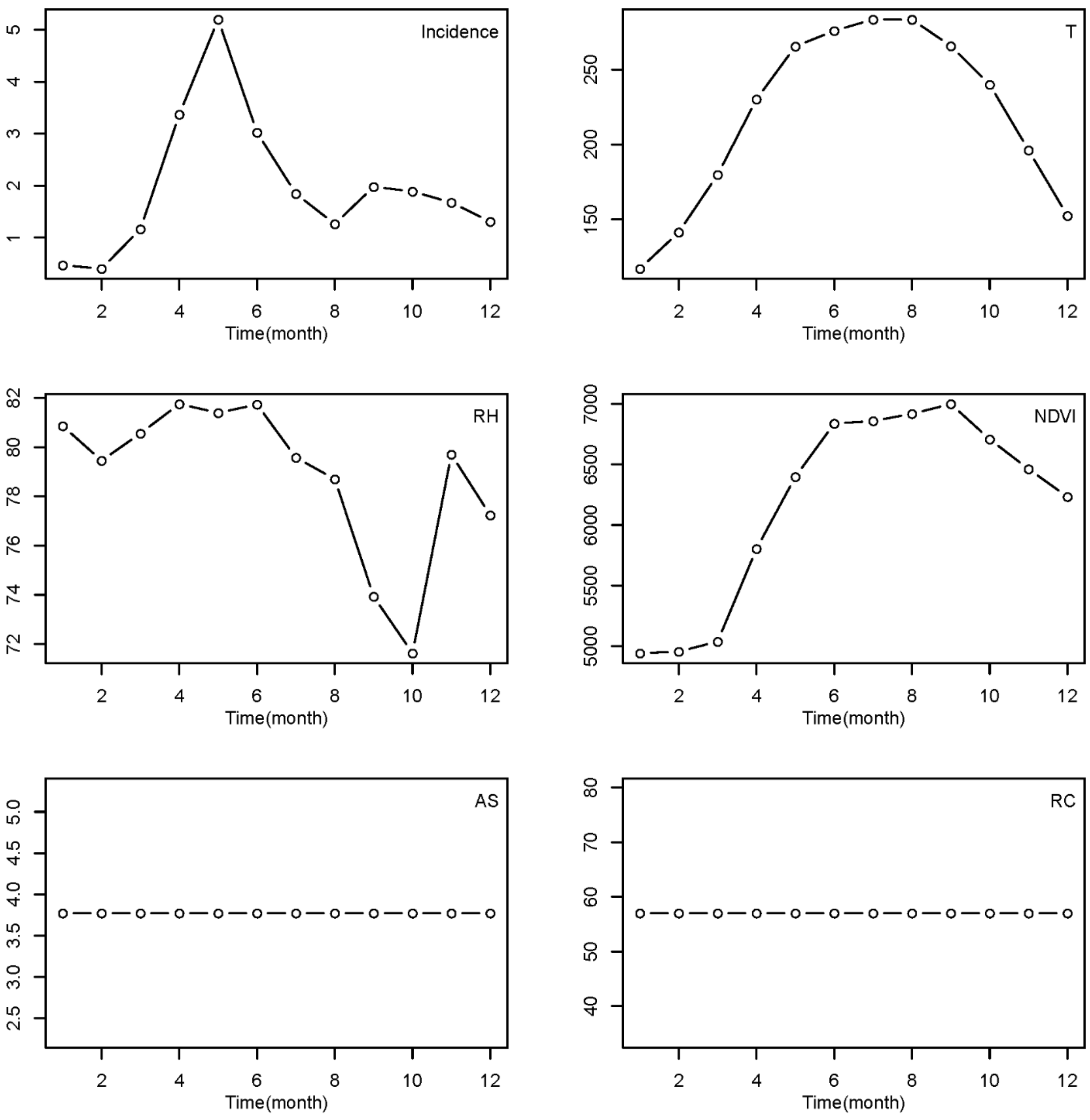

Figure S1. The temporal distributions for the median value of HFMD incidence and environmental factors.

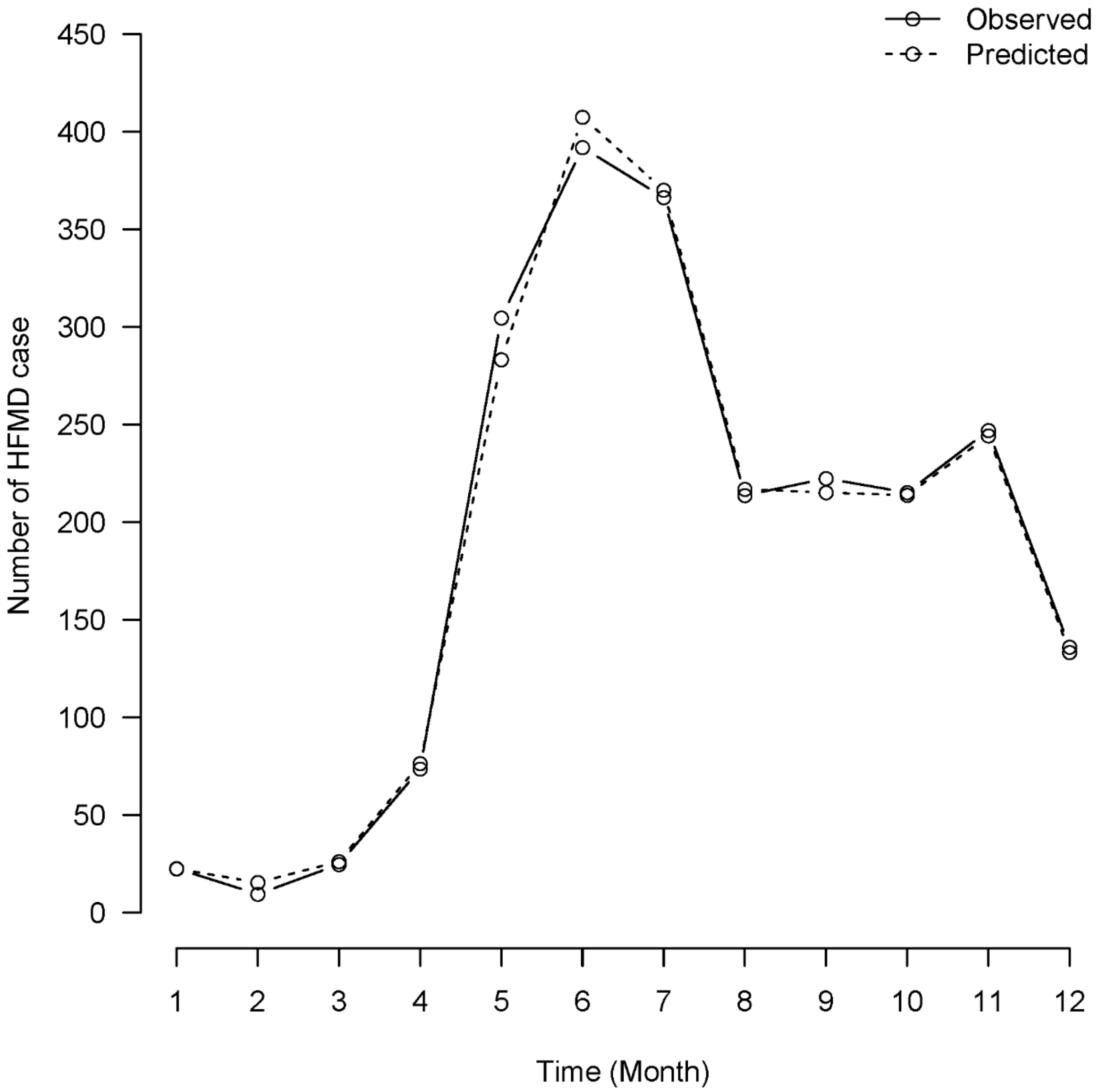

Figure S2. The observed and predicted monthly median HFMD incidence by using the final spatiotemporal Bayesian model on the testing dataset.

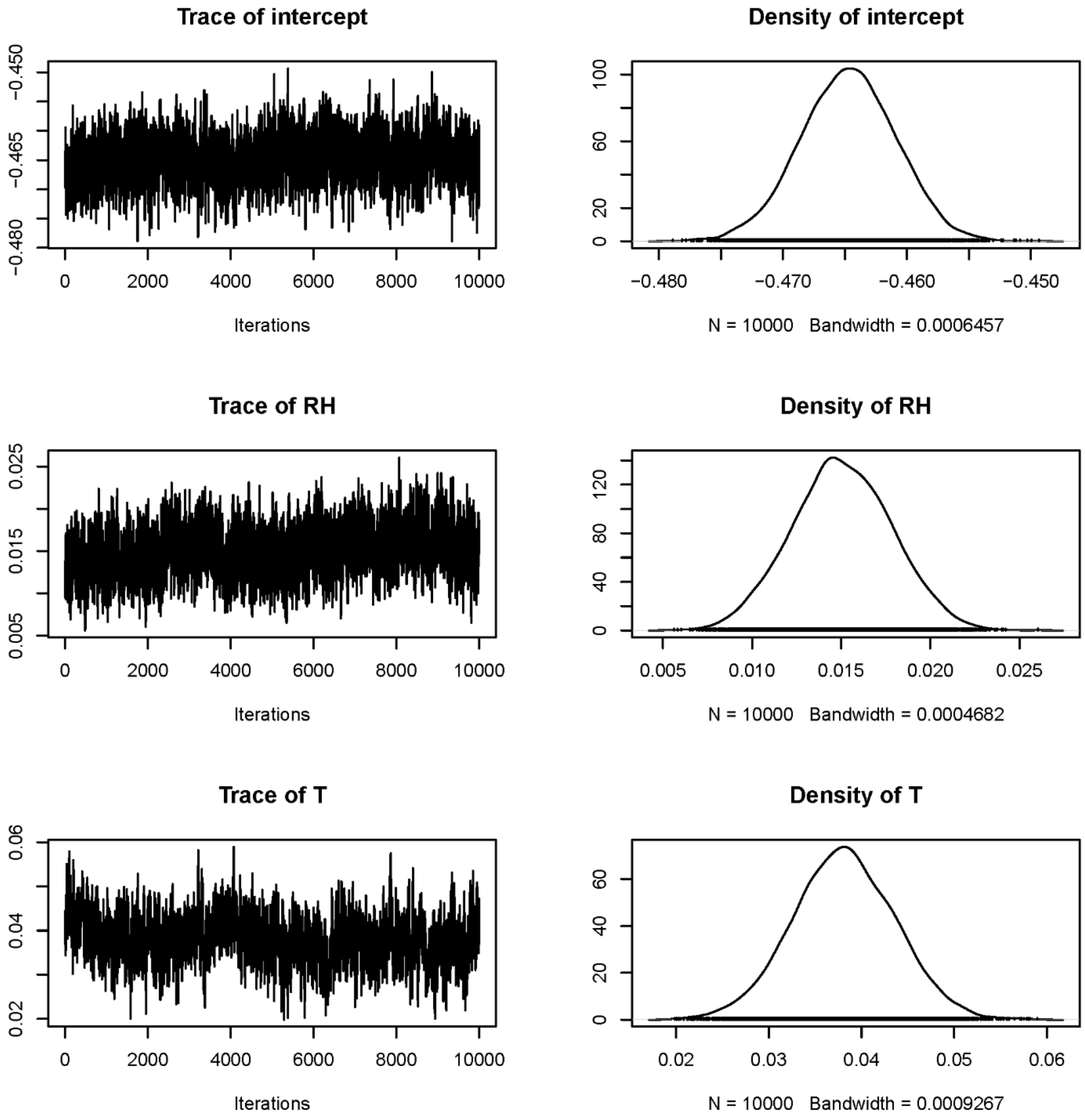

Figure S3. The trace and density from Gibbs sampling of each variable in the final model.

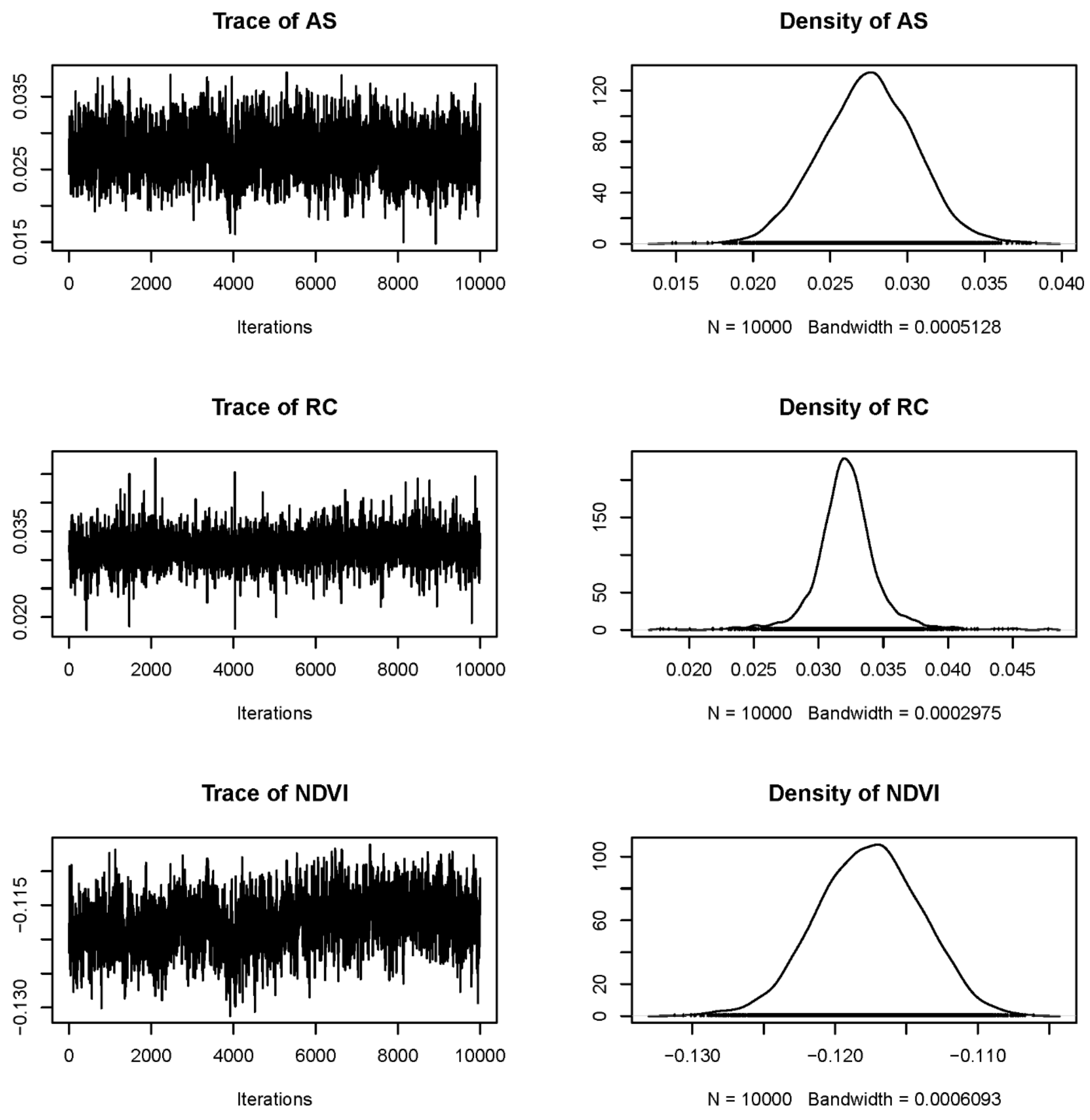

Figure S3. The trace and density from Gibbs sampling of each variable in the final model. (continuous)
